# Supplementary material for: Somatic Alteration Burden Involving Non-Cancer Genes Predicts Prognosis in Early-Stage Non-Small Cell Lung Cancer
Source: Cancers (Basel). 2019 Jul 19;11(7):1009. doi: 10.3390/cancers11071009 (PMC6678704; doi:10.3390/cancers11071009)
Supplement: Supplementary file 1 [file cancers-11-01009-s001.zip › cancers-549668-Supplementary.pdf]

Supplementary Materials: Somatic Alteration Burden Involving Non-Cancer Genes Predicts Prognosis in Early-Stage Non-Small Cell Lung Cancer

Dennis Wang, Nhu-An Pham, Timothy M. Freeman, Vibha Raghavan, Roya Navab, Jonathan Chang, Chang-Qi Zhu, Dalam Ly, Jiefei Tong, Bradly G. Wouters, Melania Pintilie, Michael F. Moran, Geoffrey Liu, Frances A. Shepherd and Ming-Sound Tsao

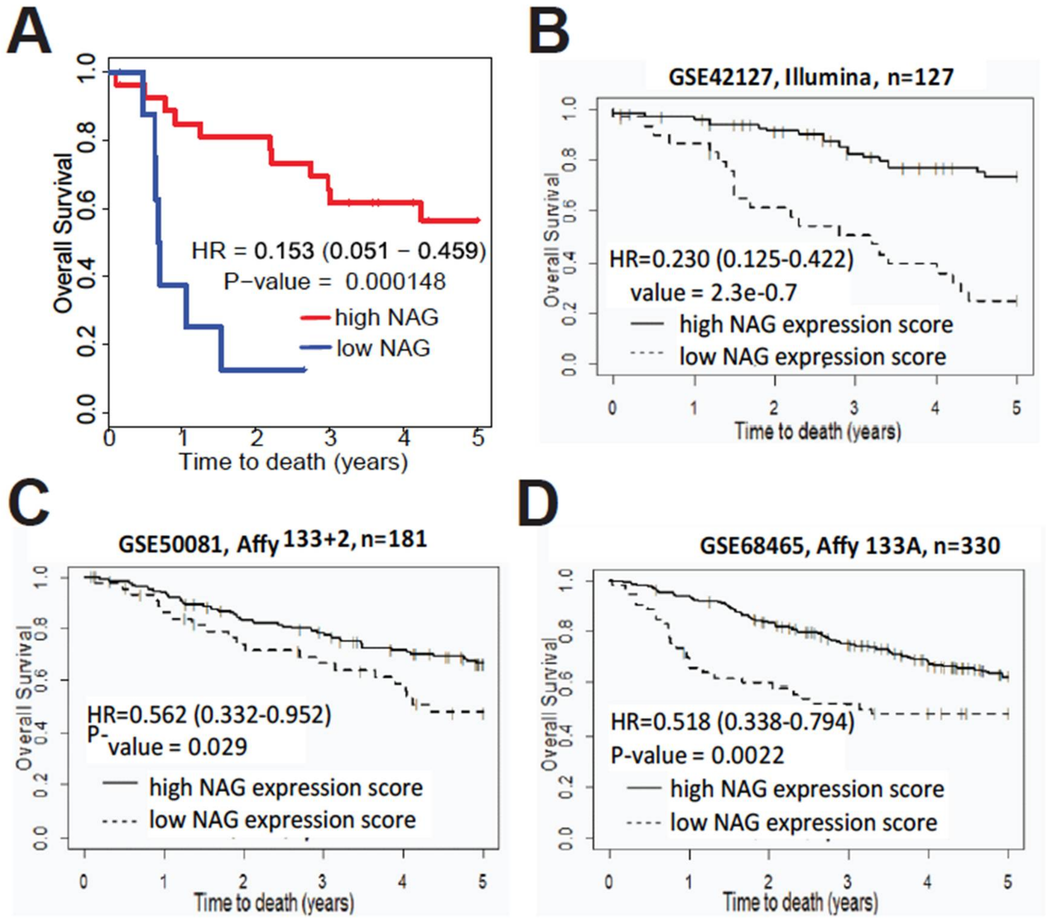

Figure S1. Association between number of altered genes and overall survival in four independent cohorts. (**A**) Kaplan-Meier curves show overall survival for the corresponding patients of the PDXs, stratified by the number of somatically altered genes (NAG). Differentially expressed genes between high and low NAG groups of NSCLC patients (**B**) were used to generate an expression score that was tested for stratifying patient prognosis in two independent NSCLC patient cohorts (**C**,**D**). Illumina RNAseq was used to quantify expression in study GSE42127, while Affymetrix microarray were used to quantify expression in GSE50081 and GSE68465.

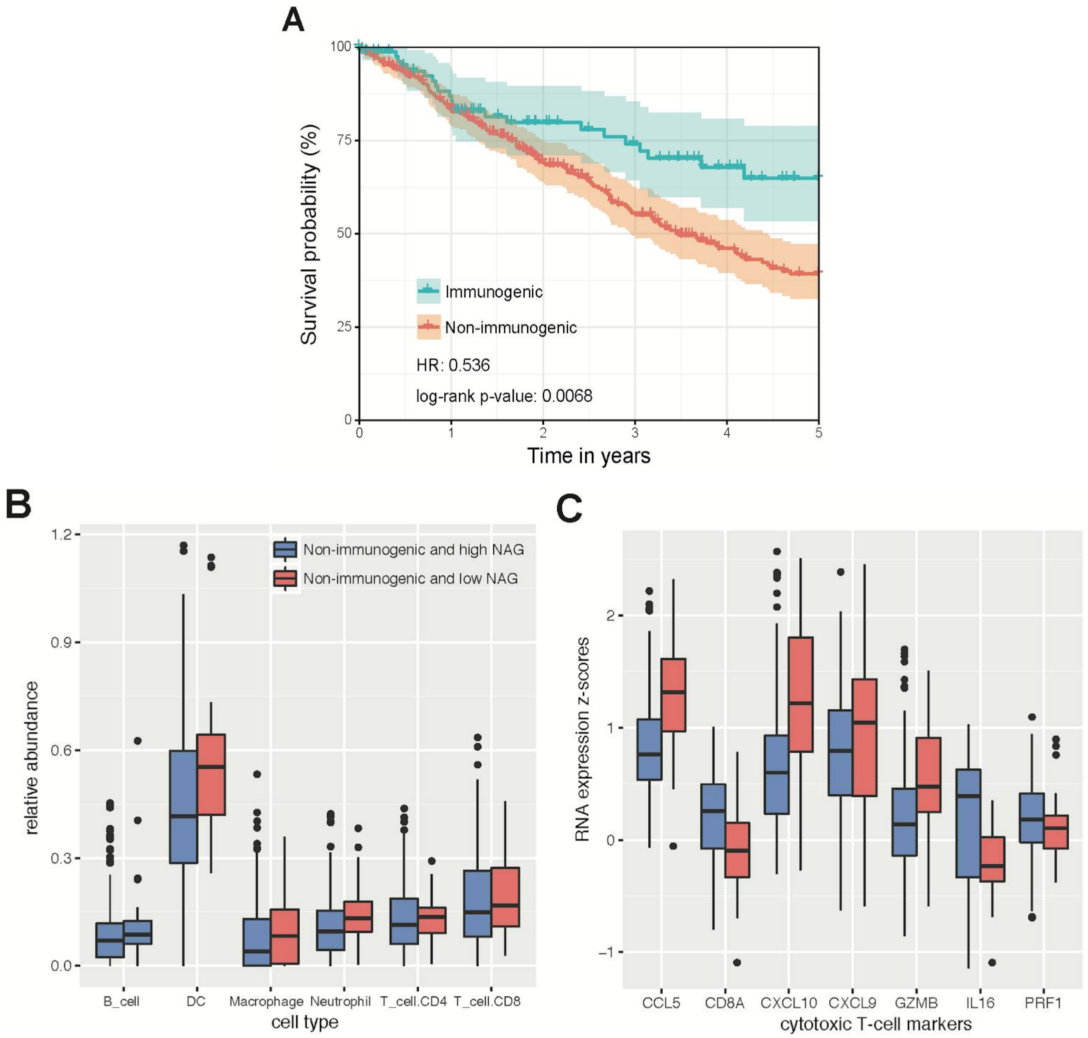

Figure S2. Effect of immunogenic peptides in stage I patients. (**A**) Overall survival of 86 TCGA NSCLC patients with immunogenic neoantigens (teal) and 310 patients without immunogenic peptides (red). Patients without immunogenic peptides stratified into high and low NAG groups are contrasted by their relative abundance of immune cell types estimated by the TIMER algorithm (**B**) and the RNA expression of cytotoxic T-cell markers (**C**).

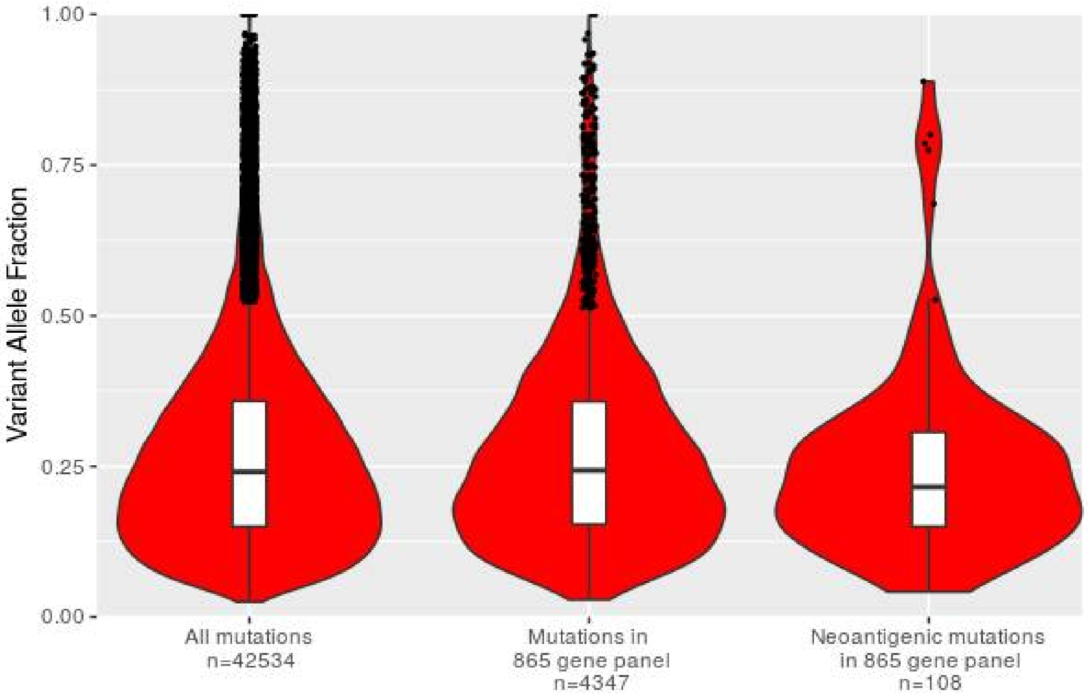

Figure S3. Distributions of variant allele frequencies (VAFs) of missense mutations used to stratify the TCGA NSCLC stage I patients. Three categories of mutations are included: all missense mutations, missense mutations in the 865 gene panel only, and immunogenic missense mutations in the 865 gene panel. Boxplots show the interquartile ranges and outliers are plotted as separate points. VAFs for all 3 groups were not significantly different between the three groups of mutations.

Tables S1–S4 are in a separated Excel file.

Table S5. Survival analysis of neoantigens estimated from all coding mutations.

Threshold	Cut-Off for Number of Neoantigen	Patient Number < Cut-Off	Patient Number ≥ Cut-Off	Hazard Ratio (95% Confidence Interval)	Long-Rank *p* Value
Median	1	132	86	0.487 (0.281–0.844)	0.0104
75%	3	169	49	0.369 (0.169–0.809)	0.0128
90%	8	196	22	0.545 (0.171–1.74)	0.305

Table S6. Clinical data from TCGA NSCLC stage I patients across subpopulations.

Variation	Low NAG	High NAG	High NAG + Neoantigen	All
Number of Patients	33	147	37	217
Age (Median)	71	68	66	68
Female	8 (24%)	77 (52%)	17 (46%)	102 (47%)
Male	25 (76%)	70 (48%)	20 (54%)	115 (53%)
Stage IA	7 (21%)	59 (40%)	12 (32%)	78 (36%)
Stage IB	26 (79%)	87 (59%)	24 (65%)	137 (63%)
Adenocarcinoma	0 (0%)	95 (65%)	25 (68%)	120 (55%)
Squamous cell	33 (100%)	52 (35%)	12 (32%)	97 (45%)
Non-smoker (Lifelong)	0 (0%)	17 (12%)	3 (8%)	20 (9%)
Adjuvant Therapy	0 (0%)	5 (3%)	3 (8%)	8 (4%)
Survival past 5 years	16 (48%)	100 (68%)	34 (92%)	150 (69%)

NAG number of altered genes

Table S7 is in a separated Excel file.

© 2019 by the authors. Licensee MDPI, Basel, Switzerland. This article is an open access article distributed under the terms and conditions of the Creative Commons Attribution (CC BY) license (http://creativecommons.org/licenses/by/4.0/).
